# Hypoxia drives CBR4 down‐regulation promotes gastroenteropancreatic neuroendocrine tumors via activation mammalian target of rapamycin mediated by fatty acid synthase

**DOI:** 10.1002/ccs3.12041

**Published:** 2024-06-22

**Authors:** Mujie Ye, Lin Xu, Feiyu Lu, Lingyi Chen, Chunhua Hu, Jinhao Chen, Bingyan Xue, Danyang Gu, Ruitong Xu, Yanling Xu, Ping Yu, Yan Wang, Ye Tian, Guoqin Zhu, Qiyun Tang

**Affiliations:** ^1^ Department of Geriatric Gastroenterology Neuroendocrine Tumor Center Jiangsu Province Hospital The First Affiliated Hospital of Nanjing Medical University Institute of Neuroendocrine Tumor Nanjing Medical University Nanjing China; ^2^ Digestive Endoscopy Jiangsu Province Hospital The First Affiliated Hospital of Nanjing Medical University Nanjing China; ^3^ Department of Gastroenterology The Friendship Hospital of Ili Kazakh Autonomous Prefecture Ili & Jiangsu Joint Institute of Health Yining China

**Keywords:** *CBR4*, everolimus, *FASN*, gastroenteropancreatic neuroendocrine tumors, hypoxia, lipids metabolism

## Abstract

Hypoxia has been highly proven a hallmark of tumor micro‐environment, promoting the malignant phenotypes, playing a crucial role from tumor initiation, progression, invasion, and intravasation to metastatic dissemination and outgrowth. Increasing evidence also showed that hypoxia mediated the abnormal lipid metabolism in cancer by regulating various oncogenic signal pathways. However, it is still unclear but attractive how hypoxia specifically functioned and changed the condition of the tumor micro‐environment. In present study, we find that hypoxia promoted the methylation degree of *CBR4* promoter region thus downgraded the expression of *CBR4*, which promoted GEP‐NETs progression and increased the sensitivity of GEP‐NETs cells to everolimus. Further, CBR4 interacted with fatty acid synthase (FASN), displaying a down‐regulation of *FASN* by activating the ubiquitin proteasome pathway and suppressed mTOR signaling. Overall, our results uncovers the *CBR4/FASN/mTOR* axis as a mechanism for tumor development and inspires us a new molecular guide for the therapeutic strategies for GEP‐NETs treatment.

## Graphical abstract

Hypoxia promotes the progression of GEP‐NETs and reduces the sensitivity to everolimus by CBR4/FASN/mTOR axis which provide a new inspiration for GEP‐NETs treatment.

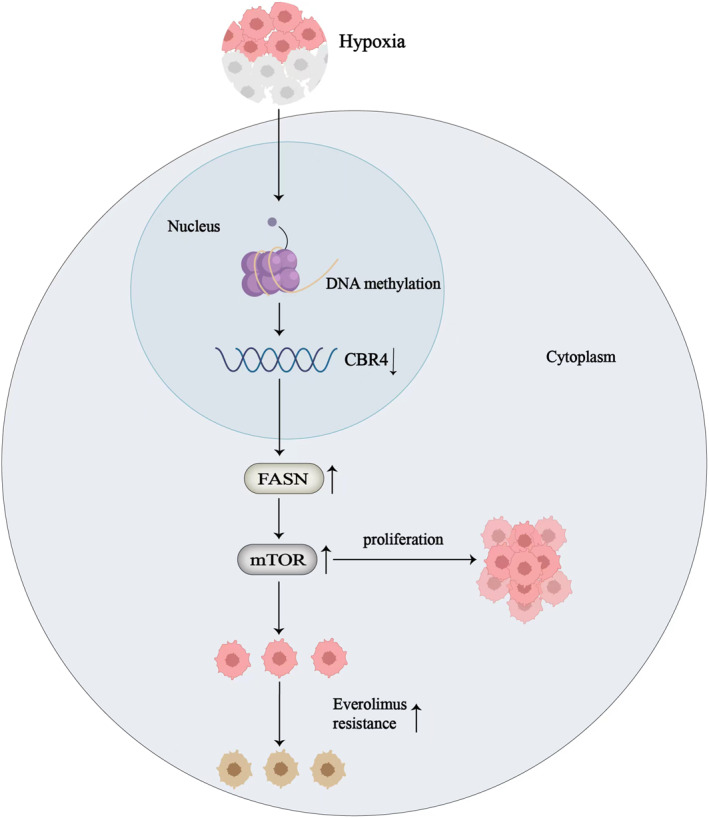

## 1 INTRODUCTION

Gastroenteropancreatic neuroendocrine tumors (GEP‐NETs) are heterogeneous malignancies arising from the neuroendocrine cells, which locate on the gastro‐intestinal tract (GI‐NETs) and the pancreas (pNETs).^1^ The incidence of this rare tumor has greatly increased in the last decade due to improved awareness and diagnostic techniques.^2^ However, the overall survival of GEP‐NETs is not ideal because the current understanding of the molecular biology of GEP‐NETs is still not sufficient to improve curative effect.

Beyond the genetics and epigenetics, recent sight of tumor has transferred towards the tumor micro‐environment(TME), which featured with hypoxia.^3^ Hypoxic micro‐environment influences tumor cells in several aspects like genome instability, autophagy, metabolic reprogramming, angiogenesis, migration, invasion, extracellular matrix remodeling, epithelial mesenchymal transition, stem cell maintenance, and immune evasion.^4^

Cancer cells regulate lipid metabolism, such as lipid uptake, synthesis, and hydrolysis, to support their rapid proliferation, survival, migration, invasion and metastasis in TME.^5^ Numerous envidences have shown that hypoxia‐inducible factor 1α (HIF‐1α) is central to the regulation of lipid metabolism.^6^ And the interaction between the altered lipid metabolism with the TME drives the cancer to a malignant phenotype through lipid mediators such as prostaglandin E2 (PGE2) and lysophosphatidic acid (LPA).^7^

In the present study, hypoxia promoted the progression of GEP‐NETs and reduced the sensitivity to everolimus by downgraded the expression of *CBR4*. And further results prove the anti‐tumor status of *CBR4* in the GEP‐NETs by decreasing the expression of *FASN* in an ubiquitin proteasome manner, which might provide a new inspiration for treatment.

## 2 METHODS

### 2.1 Cell lines and cell culture treatments

The human pNETs‐derived QGP‐1 cell line has been registered with the Japanese Collection of Research Bioresources (JCRB) cell bank (JCRB0183). The STC‐1 cell line was purchased from the ATCC (CRL‐3254). QGP‐1 cell lines were maintained in RPMI‐1640 medium (Gibco), STC‐1 cells were maintained in DMEM (Gibco), supplemented with 10% fetal bovine serum(FBS, Yeasen Biotechnology, Shanghai, China) and 1% penicillin–streptomycin (New Cell & Molecular Biotech, Suzhou, China) in a cell incubator under 5% CO2 at 37°C. To explore the effects of hypoxia on the progression of GEP‐NETs, we treated QGP‐1 and STC‐1 cells in a hypoxic cell incubator with 1% O2 for 24 h.

### 2.2 Quantitative real time‐polymerase chain reaction (qRT‐PCR) and RNA‐seq

Total RNA was isolated from cells using the trizol reagent (Vazyme, Nanjing, China) and quantified by a Nanodrop 2000. Subsequently, RNA was used to synthesize cDNA using 4xHifair® III SuperMix plus (Yeasen) according to the manufacturer's protocols. Real‐time PCR was then conducted with ChamQ Universal SYBR qPCR Master Mix. Primer information was listed in Table S1. For RNA‐seq, Total RNA was obtained from QGP‐1 cells treated with hypoxia for 24h and control groups with TRIzol® (Takara Bio, Inc.).RNA samples were sequenced by Hangzhou Lianchuan Biotechnology Co., Ltd.

### 2.3 Western blotting (WB)

Total proteins from cells were lysed with RIPA buffer containing a protease inhibitor (Beyotime, Nantong, China) and boiled with 1 × loading buffer. The extracted samples were separated by 10% sodium dodecyl sulfate–polyacrylamide gel (SDS‐PAGE) electrophoresis and transferred onto a nitrocellulose filter membrane. The membranes were blocked using 8% skim milk and then washed three times with TBST. The blocked membranes were incubated with primary antibodies overnight at 4°C, followed by secondary antibodies (CWBIO, Taizhou, China) at room temperature for 2 h. Antibody information was listed in Table S2. The signals were developed using the image lab software with an enhanced chemiluminescence substrate(New Cell & Molecular Biotech).

### 2.4 Construction of stably transfected cell lines

*CBR4* knockdown and over‐expression plasmids were respectively constructed in the PLKO1 and PLVX vector both by Genomeditech(Shanghai, China). The short hairpin targets of CBR4 were listed in Table S3. And plasmids transfection was accomplished in 293T cells using PEI MAX(Polysciences, USA) with serum‐free medium for 6h, followed by the addition of the corresponding serum. Then we harvested the viral supernatant through a 0.45‐μm filter after 48 h and applied it to cells having 50% confluence. Finally, 2 μg/mL puromycin was used for 7 days to select stable cell lines.

### 2.5 Co‐immunocoprecipitation

1 mL of RIPA lysate was added to a 10‐cm dish, and protein was extracted by conventional methods. After incubation with 5  μg antibody for 2 h at 4°C, 50  μL protein A/G magnetic beads were added and inverted overnight. The next day samples were washed thoroughly three times with RIPA lysate (Beyotime), incubated with 30  μL 2 × SDS‐PAGE loading buffer and boiled at 100°C for 10 min before subsequent WB experiments.

### 2.6 Cell counting Kit‐8 (CCK‐8) assay

Proliferation was detected with CCK‐8 (New Cell & Molecular Biotech). In brief, 5 × 10^3^ cells per well were cultured in 100 μL medium in 96‐well plates for 0, 24, 48, and 72 h, followed by the addition of CCK8 reagent (Yeasen) for 2 h under 37°C, and analyzed by a microplate reader at 450 nm absorbance(Thermo Fisher, USA).

### 2.7 Colony formation assay

For the colony formation assay, 1 × 10^4^ QGP‐1 and STC‐1 cells per well were seeded in each 6‐well plate cultured in medium with 10% FBS for 10 days in a constant temperature incubator. The cells were then fixed with 4% paraformaldehyde for 30 min and then stained with 0.2% crystal violet for 30 min.The number of colonies was counted by Image J.

### 2.8 5‐ethynyl‐2′‐deoxyuridine (EdU) incorporation assay

Cells already planted in the 96‐well plates were treated with 50 μM EdU medium (RiboBio, Guangzhou, China) at 37°C for 2 h, then fixed with 4% paraformaldehyde for 30 min. Next, 0.5% Triton‐X was used to permeabilize the cells and a 1 × Apollo reaction cocktail (RiboBio) was used to treat the cells for 30 min. The DNA contents of the cells were stained by treating the cells with Hoechst33342 for 30 min at room temperature.Finally, images were obtained using fluorescence microscope(Zeiss, Germany).

### 2.9 Xenograft in nude mice and immunohistochemistry

For the in vivo xenograft model, a total of 5 × 10^6^ QGP‐1cells (treated with *CBR4* knockdown or over‐expression) were suspended in 100 μL PBS and subcutaneously injected into the armpit of the pathogen‐free mice(4–6 weeks). After 4 weeks, the mice were sacrificed and the tumors were weighed, photographed, and fixed in 4% paraformaldehyde or frozen for further analysis. Tumor volume was measured by the following formula: volume = 1/2 × length × width^2^. All animal experiments were approved by the Institutional Animal Care and Use Committee (IACUC) of Nanjing Medical University.

Besides, tumor tissues were embedded in paraffin and sectioned into 5 μm slices for immunohistochemistry of Ki67, *CBR4* and *FASN*. Images were taken randomly from each slide.

### 2.10 Statistical analysis

Results were presented as mean ± SD and analyzed by GraphPad Prism 8.0 (GraphPad, Inc., USA). Student's *t*‐test was used to calculate significant differences in two‐group comparisons. All assays were independently repeated at least three times and *p* < 0.05 was reckoned as statistically significant.

## 3 RESULTS

### 3.1 Hypoxia promotes the proliferation of GEP‐NETs cells and reduces the sensitivity to everolimus

**FIGURE 1:**
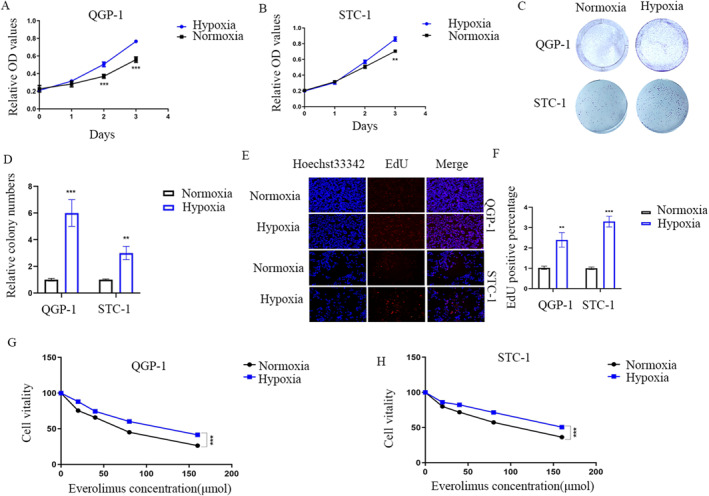
Hypoxia promotes cancer proliferation and reduces the sensitivity to everolimus. (A, B) CCK‐8 data showing that treatment of hypoxia promoted proliferation of QGP‐1 (A) and STC‐1 (B) cells. (C, D) Hypoxia treatment induced increased clone numbers. (E, F) Hypoxia promoted proliferation of QGP‐1 and STC‐1 cells, as observed with the EdU assay, magnification:×200. (G, H) CCK‐8 data displaying that hypoxia reduced the sensitivity of QGP‐1 and STC‐1 to everolimus.

To figure out what the role hypoxia play in GEP‐NETs, we treated QGP‐1 and STC‐1 cells with or without hypoxia (1% O2) for the indicated time. CCK‐8 and EdU assays were performed, which both showed the proliferation was promoted with hypoxia‐treated cells compared with the normoxia‐treated ones (Figure 1A,B, E, F). Moreover, data from the clone formation assay showed increased clone numbers in hypoxia‐treated QGP‐1 and STC‐1 cells (Figure 1C,D). As everolimus is a commonly used target drug for GEP‐NETs, which mainly inhibits the mammalian target of rapamycin (mTOR) pathway, and hypoxia is an important blasting fuse for the activation of the mTOR pathway. Another CCK‐8 assay of cells treated with everolimus in different concentrations suggested that hypoxia reduced the sensitivity of QGP‐1 and STC‐1 to everolimus (Figure 1G,H).

### 3.2 Hypoxia down‐regulated *CBR4* expression and involved in the fatty acids metabolism

**FIGURE 2:**
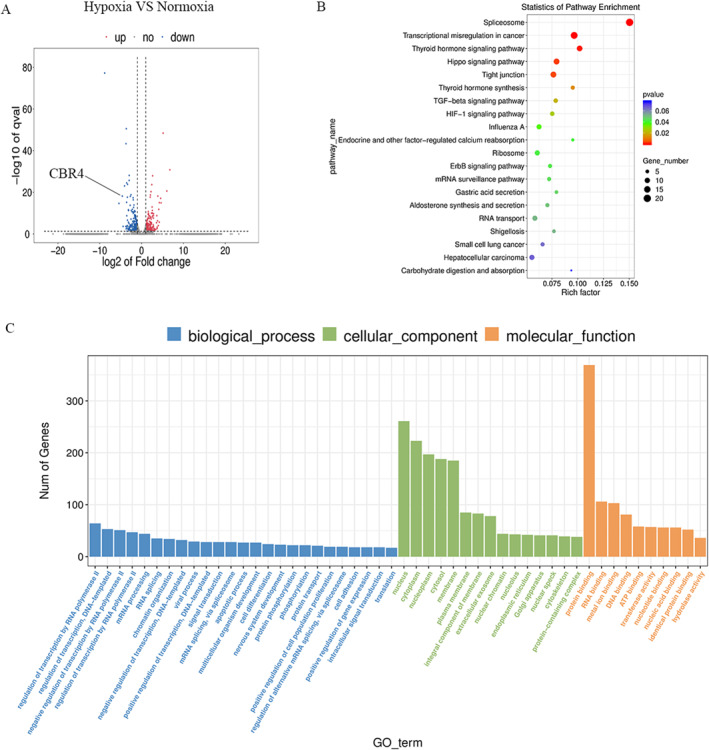
RNA‐seq assays showed the differential expression of CBR4 and high relevance of fatty acid metabolism processes. (A) CBR4 was down‐regulated by hypoxia treatment. (B) Enrichment analysis for RNA‐seq results demonstrated the pathways hypoxia involved in various pathways. (C) GO analysis for RNA‐seq results showed that hypoxia participated in fatty acid metabolism processes.

**FIGURE 3:**
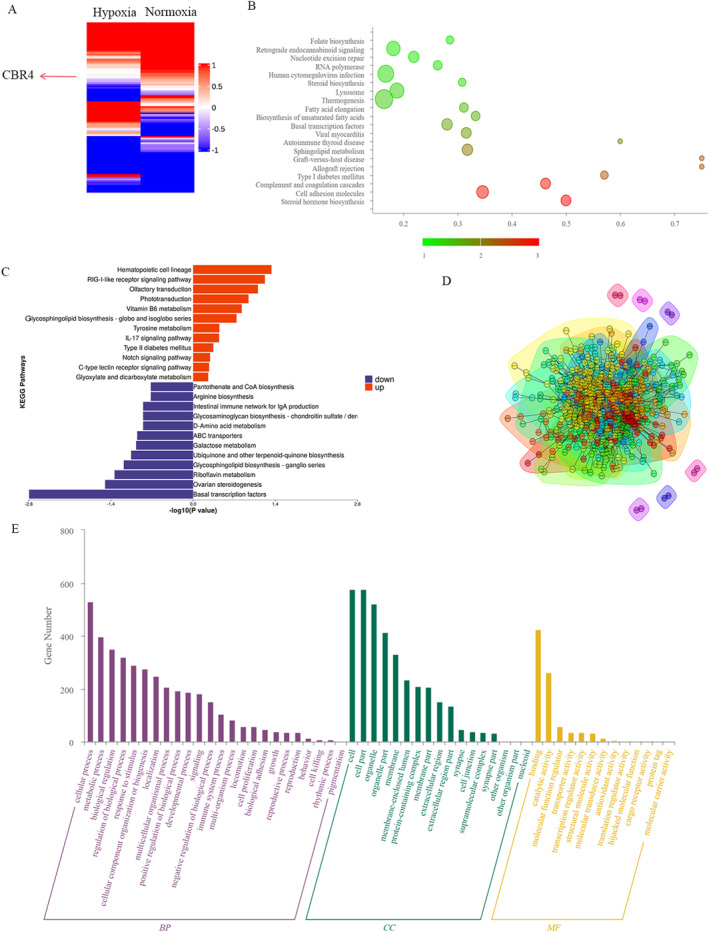
Proteomic sequencing revealed the CBR4 expression and relevant pathways from the protein perspective. (A) Heatmap showed the differential expression of CBR4. (B) The analysis of relevant pathways and disease after hypoxia treatments. (C) KEGG pathway analysis after hypoxia treatments. (D) A grid connection diagram of connections between the differential proteins. (E) GO analysis of expression of different related genes.

**FIGURE 4:**
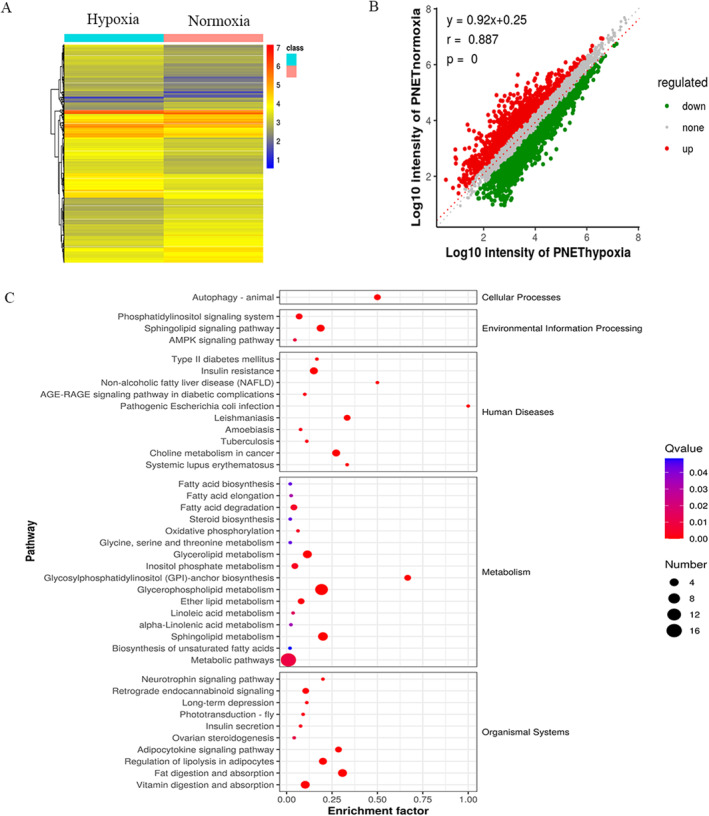
Metabolomics sequencing showed the effect of hypoxia on cellular metabolism. (A, B) Heat map and volcano map of metabolomics sequencing. (C) Enrichment analysis displayed the pathways that were up‐regulated or down‐regulated.

To study the underlying molecular mechanism of hypoxia in GEP‐NETs cells, RNA‐seq assays were conducted between QGP‐1 cells treated with hypoxia and normoxia to further find out the metabolism related molecules (Figure 2A). Among these differential genes, we found *CBR4* was significantly down‐regulated and related to lipids metabolism. KEGG pathway analysis demonstrated that hypoxia might connect to many signaling pathways including Thyroid hormone, Hippo, TGF‐*β* and HIF‐1 signaling pathway (Figure 2B). Gene Ontology(GO) analysis further showed that hypoxia might be involved in various biological processes (Figure 2C). Proteomic sequencing also revealed the differential expression of *CBR4* (Figure 3A) from the protein perspective and identified many relevant disease and pathways including fatty acid metabolism processes that were dy‐regulated after hypoxia treatment (Figure 3B,C). The connections between the differential proteins were also plotted as a grid connection diagram (Figure 3D).The GO analysis showed high expression of genes relating to metabolic process and biological regulation (Figure 3E). The heat map and volcano map of metabolomics sequencing demonstrated the significant effect of hypoxia on cellular metabolism and found it affect fatty acid degradation and glycerophospholipid metabolism,and highly associated with insulin resistance (Figure 4A,C).

### 3.3 Down‐regulation of *CBR4* promotes the proliferation of GEP‐NETs cells and reduces the sensitivity to everolimus

**FIGURE 5:**
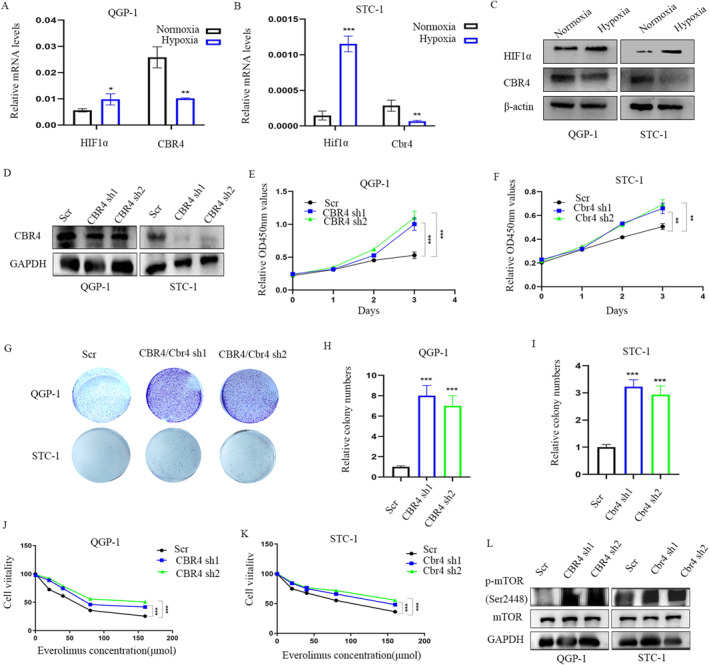
Down‐regulated CBR4 promotes the proliferation of GEP‐NETs cells and reduces the sensitivity to everolimus. (A–D) q‐PCR and western blot analysis of the RNA and protein levels of HIF‐*α* and CBR4 in hypoxia‐treated and control groups. (E, F) CCK‐8 data showing that knockdown of CBR4 promoted proliferation of QGP‐1 (E) and STC‐1 (F) cells. (G–I) Knockdown of CBR4 induced increased clone numbers. (J, K) CCK8 assays indicated CBR4 silence reduced the sensitivity of QGP‐1 (J) and STC‐1 (K) to everolimus. (L) WB analysis of the relationship of CBR4 and mTOR pathway.

To further confirm the relationship between hypoxia and *CBR4*,5 qRT‐PCR and WB of QGP‐1 and STC‐1 cells were performed to demonstrate that hypoxia down‐regulated *CBR4* expression, accompanied with an increase in HIF‐1α (Figure 5A–C).To explore the role of *CBR4* in GEP‐NETs, we constructed QGP‐1 and STC‐1 stable *CBR4* knockdown cell lines with the aid with lentivirus and the efficiency of transfection was confirmed via WB (Figure 5D). Then it obviously showed in CCK‐8 assays and colony formation assays that compared with wild strains, knockdown of CBR4 significantly increased the cell proliferation rate of GEP‐NETs (Figure 5E–I). Moreover, knockdown of *CBR4* inhibited the everolimus sensitivity in both QGP‐1 and STC‐1 cells (Figure 5J,K). And WB results showed *CBR4* has an obvious inactivation of mTOR (Figure 5L).

### 3.4 *CBR4* decrease the expression of *FASN* in an ubiquitin proteasome manner

**FIGURE 6:**
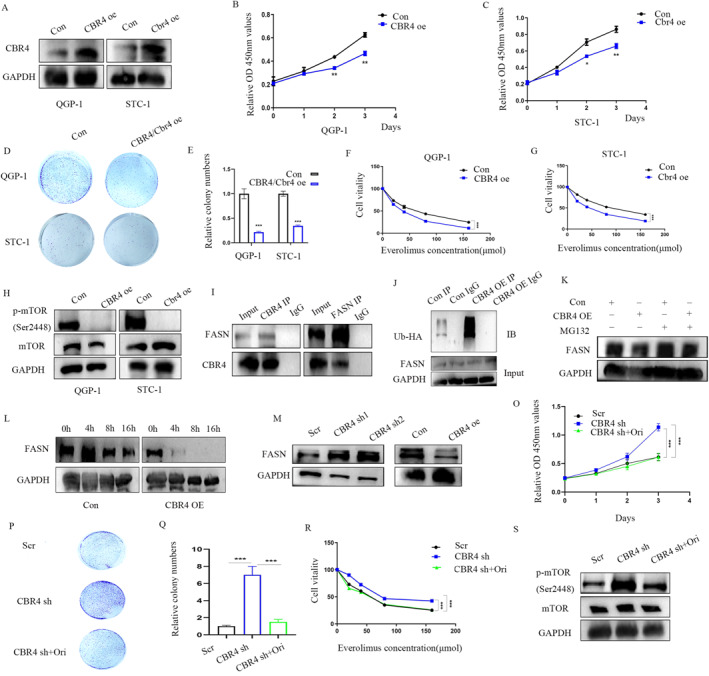
CBR4 decrease the expression of FASN via ubiquitin proteasome manner. (A) Western blot analysis of the protein levels of CBR4 in CBR4 over‐expression and control groups. (B, C) CCK‐8 assays showed the proliferation of control group and CBR4 over‐expression cells. (D, E) Over‐expression of CBR4 induced a decrease in clone numbers of QGP‐1 and STC‐1 cells (E). (F, G) CCK8 assays indicated CBR4 over‐expression induced the sensitivity of QGP‐1 (F) and STC‐1 (G) to everolimus. (H) WB analysis of the expression of mTOR in control, CBR4 over‐expression groups. (I–K) The Co‐IP assays showed the interactions between CBR4 with FASN (I) and an up‐regulation in ubiquitin of FASN in CBR4 over‐expression group (J), which was reserved with the treatment of MG132 (K). (L) WB analysis showed the level of FASN at different points in CBR4 over‐expression and control groups after treated with CHX. (M) The FASN expression in CBR4 over‐expression and knockdown group was also displayed. (O, P, Q) Knockdown of CBR4 induced an increase of cell proliferation that could be reserved by the addition of Orlistat in CCK‐8 and colony assays. (R) CCK8 assays indicated Orlistat reduced the resistant to everolimus caused by CBR4 knockdown in QGP‐1 cells. (S) WB analysis of the expression of mTOR in control, CBR4 knockdown with or without Orlistat treatment groups.

To ascertain whether *CBR4* plays a tumor suppressor role in GEP‐NETs, we generated stable CBR4 over‐expressing QGP‐1 and STC‐1 cell lines via stable transfection. And the over‐expression efficiency were validated in the WB results (Figure 6A). The cell proliferation was suppressed in the *CBR4*‐expressing QGP‐1 and STC‐1 cells as it showed in the CCK‐8 assays (Figure 6B,C).The inhibition was also verified from the clone formation assays (Figure 6D,E).The CCK‐8 assays of QGP‐1 and STC‐1 cells treated with different concentration of everolimus displayed that *CBR4*‐expressing increased the sensitivity of GEP‐NETs to everolimus (Figure 6F,G).WB result showed that the over‐expression of *CBR4* led to the inactivation of mTOR (Figure 6H). As suggested by the above multi‐omics analysis that hypoxia effect the fatty acids metabolism with *CBR4* downgraded, the subsequent step involved an investigation into the correlation between *CBR4* and *FASN*, which was a fatty acid synthase.The Co‐IP assays confirmed interactions between *CBR4* with *FASN* (Figure 6I). Co‐IP assay of ubiquitin showed that over‐expression of CBR4 caused an up‐regulation in ubiquitin combined with FASN compared to the control groups (Figure 6J). Moreover,the expression of *FASN* was reserved in the *CBR4* over‐expression group with the treatment of MG132 (Figure 6K). After treatment with CHX, the stability of FASN was decreased at different points in CBR4 over‐expression groups (Figure 6L).The expression of FSAN was inhibited in the *CBR4* over‐expression cells while an opposite result in *CBR4* knockdown cells (Figure 6M).To further explore the role of *FASN* regulated by *CBR4* in the development of GEP‐NETs, we performed a series of rescue phenotypes. The result of CCK‐8 and colony formation assays revealed that the incerased proliferation ability of *CBR4* deficiency cells was reversed by the addition of orlistat, which is a *FASN* inhibitor (Figures 6O‐6Q). Furthermore, the high resistant to everolimus of *CBR4* knockdown cells was also reversed by orlistat (Figure 6R). And *CBR4* deficiency cells treated with orlistat, the mTOR signal was inhibited compared to the *CBR4* knockdown group (Figure 6S).

### 3.5 Hypoixia induced low expression of *CBR4* via increased the methylation degree of CBR4

**FIGURE 7:**
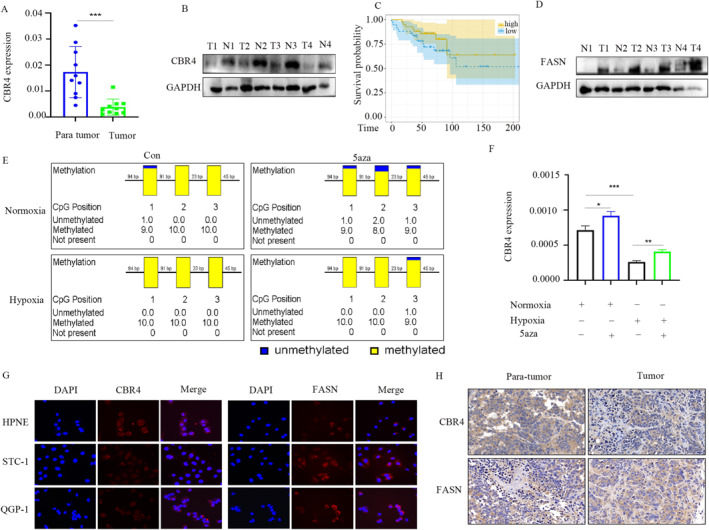
High methylation degree of CBR4 leads to low expression of CBR4. (A, B) q‐PCR analysis of CBR4 expression and WB analysis of the expression of CBR4 between pNETs tissue and normal tissue adjacent to pNETs. (C) Kaplan‐Meier analysis of samples divided into CBR4‐low‐expression and CBR4‐high‐expression groups based on gene expression. (D) WB analysis of the expression of FASN between pNETs tissue and normal tissue adjacent to pNETs. (E, F) The analysis of the methylation degree of CBR4 in hypoxia‐treated, control and 5‐aza treated groups. (G) Immunofluorescence of CBR4 and FASN in HPNE, QGP‐1 and STC‐1 cell lines. (H) Immunohistochemistry of the expression of CBR4 and FASN in pNETs and normal peri‐tumor tissues.

We performed qRT‐PCR and WB to compare the expression of *CBR4* between pNETs tissue and normal tissue adjacent to pNETs. The results indicated that *CBR4* appeared lower levels in pNETs tissues (Figure 7A,B). The samples were divided into *CBR4*‐low‐expression and *CBR4*‐high‐expression groups based on gene expression to perform Kaplan‐Meier analysis, which were extracted from the public database.The results showed that the pNETs patients with high *CBR4* expression had higher overall survival than those with low *CBR4* expression (Figure 7C). Another WB assays showed that the expression of *FASN* were higher in pNETs tissue, contrast to the *CBR4* (Figure 7D). The following analysis indicated that 5‐azacytidine (5‐aza) treatment, a DNA demethylation agent, led to the demethylation of *CBR4* and increased the expression, while hypoxia promoted the methylation degree of *CBR4* and decreased the expression in both control and 5‐aza treated group (Figure 7E,F). And immunofluorescence showed the location of *CBR4* and *FASN* in GEP‐NETs cell lines, and lower levels in tumor cells than normal cells (Figure 7G). Furthermore, we performed immunohistochemistry and the results revealed that *CBR4* expressed lower in pNETs tissues than in normal peritumor tissues but *FASN* presented the opposite expression (Figure 7H).

### 3.6 *CBR4* plays anti‐cancer roles in vivo

**FIGURE 8:**
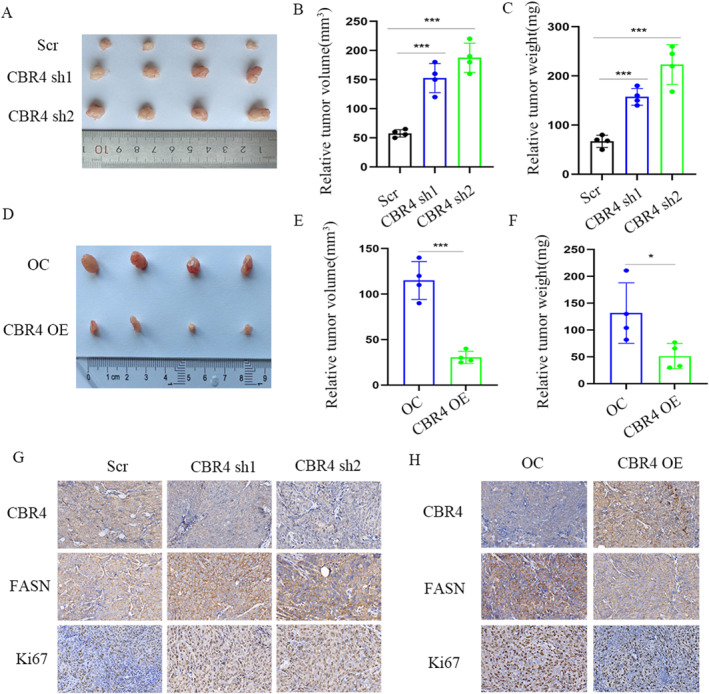
CBR4 is a tumor suppressor in vivo. (A–C) Primary tumor samples obtained from mice subcutaneously injected with QGP‐1 cells transfected with CBR4 knockdown and control cell groups (A). Relative tumor volumes (B) and weights (C) at the endpoint (*n* = 4). (D–F) Primary tumor samples were obtained from mice subcutaneously injected with QGP‐1 cells transfected with CBR4 over‐expression and control group (D). Relative tumor volumes (E) and weights (F) measured at the endpoint (*n* = 4). (G, H) Representative immunohistochemistry images showing expression of CBR4, FASN and ki67 in xenograft tumor tissues, magnification: ×73. (**p < 0.01, ***p < 0.001).

We examined the roles of *CBR4* in vivo by building tumor xenograft models.The QGP‐1 cells respectively transfected with *CBR4* knockdown,*CBR4* over‐expression vector and control vector were implanted in nude mice. Notably, the weights and volumes of tumors were increased in mouse xenografts injected with QGP‐1 cells bearing *CBR4* knockdown vectors (Figure 8A,C). Conversely, QGP‐1 cells with stable CBR4 over‐expression decelerated tumor growth compared with the control group (Figure 8D,F). The immunohistochemical staining of CBR4 and FASN revealed *CBR4* also downdraded *FASN* expression in vivo. And Ki67 expression levels were obviously lower in *CBR4* over‐expression tumors while higher in CBR4 knockdown tumors, which further proved that *CBR4* is a tumor suppressor (Figure 8G,H).

## 4 DISCUSSION

The relationship between hypoxia and lipid metabolism in tumors has been extensively studied, revealing complex mechanisms by which hypoxic conditions influence lipid metabolic reprogramming to support tumor growth and survival. Hypoxia in the tumor micro‐environment leads to up‐regulation of lipid metabolism‐related genes, such as ACSS2, which enhances lipid metabolism reprogramming through pathways like HMGCS1 mediated PI3K/AKT/mTOR, promoting the progression of various cancers, including pNETs.^8^ Current research focuses on understanding these adaptive mechanisms in detail, aiming to identify new therapeutic targets within lipid metabolic pathways to inhibit tumor growth and improve the efficacy of existing cancer treatments. The modulation of lipid metabolism in response to hypoxia represents a critical survival strategy for tumor cells, highlighting the importance of this area for potential cancer therapeutics development.

Located in the mitochondria, *CBR4* is a mitochondrial NADPH‐dependent reductase for o‐ and p‐quinones involved in fatty acid biosynthetic process; glycoside metabolic process; and protein tetramerization.^9^ As a member of the short‐chain dehydrogenase/reductase (SDR) family, along with other NAD(P) (H)‐dependent enzymes, it plays physiological roles in the metabolism of steroid hormones, prostaglandins, carbohydrates and retinoids, as well as in the metabolism of xenobiotics, drugs and carcinogens. Although there was a study found that *CBR4* may protect cells against cytotoxicity of exogenous quinones and participate in key steps of the apoptotic signal cascade induced by oxidative stress,^10^ the mechanisms of how *CBR4* functions in cells have been poorly explored.

In our experiment, *CBR4* was found to interact with fatty acid synthase (*FASN*), which further explained its involvement in cellular fatty acid metabolism. *FASN* is a multi‐enzyme protein, which is a key regulator of lipid metabolism, especially fatty acid synthesis. It can regulate the fatty acid synthesis of cancer cells or drive abnormal lipogenesis of cancer cells, and is considered to be a oncogenic factor as often found over‐expressed in cancer cells. Up‐regulation of *FASN* expression has been found to be associated with cancer progression in several cancer types, such as prostate cancer, ovarian cancer, breast cancer and liver cancer. In some cases, studies have identified *FASN* as a potential prognostic or therapeutic target using gene expression analysis.^11^ Furthermore,our previous studies revealed that *FASN* mRNA and protein expression levels were significantly higher in pNETs cells, which may due to the regulation by FABP5 via mTOR and Wnt/β‐catenin pathway.^12^, ^13^

Orlistat, beyond known for its traditional role in obesity management by inhibiting lipase, has shown promise as a cancer treatment agent due to its effect on *FASN*.^14^ Research has highlighted its potential to inhibit tumor growth, induce apoptosis in various cancer cell types, and enhance the efficacy of existing chemotherapy agents. Notably, orlistat's multitargeted mechanism, including its "dirty" drug aspect with actions on several cancer‐relevant targets, supports its repurposing for cancer therapy.^15^ In our application, orlistat was found to inhibit cell proliferation, migration and invasion by specifically inhibiting *FASN* on *CBR4* knockdown cells, and reversed the cancer‐promoting effect of *FASN*. The same results have been shown in vitro.There were studies suggesting its synergistic effects with other drugs, enhancing therapeutic outcomes in colorectal cancers,^16^ breast cancers,^17^ and ovarian cancers,^18^ among others.^19^ This evolving research underscores orlistat's potential as a versatile and effective agent in cancer treatment strategies.

Everolimus is an mTOR inhibitor extensively used in the treatment of cancers, showing significant efficacy in improving progression‐free survival among patients.^20^ Its application also spans various NET types, including pancreatic, gastrointestinal, and lung NETs, underpinning its versatility in targeting the mTOR pathway, which is crucial in NETs development. Drug resistance to everolimus emerges through various mechanisms, including alterations in the PI3K/AKT/mTOR pathway and compensatory activation of alternative survival pathways.^21^ Combining everolimus with other agents, such as somatostatin analogs or targeted therapies, has been investigated to overcome resistance and improve therapeutic outcomes. Studies have demonstrated that these combinations can lead to improved efficacy without substantially increasing toxicity, offering a strategic approach to enhance the management of advanced NETs. This evidence supports the role of everolimus as a cornerstone in the treatment of NETs, with ongoing research focusing on optimizing combination therapies to circumvent resistance and improve patient outcomes.^22^

In conclusion, our findings demonstrated that hypoxia induced the deficiency of *CBR4*,which could promote the progress of GEP‐NETs in both vivo or vitro and improve the everolimus resistance of tumor cells by decreasing the expression of *FASN* in a ubiquitin‐dependent way and activating the mTOR pathway. Furthermore, we found that orlistat might reverse the tumor suppression effect of *CBR4*. Our study not just identified a potential early diagnostic biomarker for GEP‐NETs but provided a promising combination of orlistat and everolimus in the treatment of GEP‐NETs.

## AUTHOR CONTRIBUTIONS

**Qiyun Tang, Ye Tian** and **Guoqin Zhu**: Conceptualization, methodology, software; **Mujie Ye, Lin Xu, Feiyu Lu, Lingyi Chen, Chunhua Hu** and **Jinhao Chen**: Data curation, writing‐original draft preparation; **Bingyan Xue, Danyang Gu, Ruitong Xu, Yanling Xu, Ping Yu**: Visualization, investigation; **Qiyun Tang** and **Mujie Ye**: Writing‐reviewing and editing; **Chunhua Hu, Lin Xu, Feiyu Lu** and **Ping Yu**: Software, validation; **Qiyun Tang, Guoqin Zhu** and **Ye Tian**: Supervision. All authors read and approved the manuscript.

## CONFLICT OF INTEREST STATEMENT

The authors have declared that no competing interest exists.

## CONSENT FOR PUBLICATION

Not applicable.

## ETHICS STATEMENT

Animal study was approved by Institutional Animal Care and Use Committee (IACUC) of Nanjing Medical University. The discarded tumor tissue was obtained with patients inform and was approved by The First Affiliated Hospital of Nanjing Medical University (Jiangsu Province Hospital).

## Supporting information

Supporting Information S1

## Data Availability

All data generated or analyzed during this study are included in this published article.
