# Supplementary material for: Hypoxia drives CBR4 down‐regulation promotes gastroenteropancreatic neuroendocrine tumors via activation mammalian target of rapamycin mediated by fatty acid synthase
Source: J Cell Commun Signal. 2024 Jun 22;18(3):e12041. doi: 10.1002/ccs3.12041 (PMC11544642; doi:10.1002/ccs3.12041)
Supplement: Supplementary file 1 — Supporting Information S1 [file CCS3-18-e12041-s001.docx]

**Supplementary Table1 Primers of genes**

| **Gene names** | **Sequence(5’-3’)** |
| --- | --- |
| HIF1α forward | GAACGTCGAAAAGAAAAGTCTCG |
| HIF1α reverse | CCTTATCAAGATGCGAACTCACA |
| CBR4 forward | GGCGGTCATTGCCAGAAAC |
| CBR4 reverse | GCAACATCACAGCTAAATGCCA |
| ACTB forward | CATGTACGTTGCTATCCAGGC |
| ACTB forward | CTCCTTAATGTCACGCACGAT |
| Hif1α forward | TCTCGGCGAAGCAAAGAGTC |
| Hif1α reverse | AGCCATCTAGGGCTTTCAGATAA |
| Cbr4 forward | AAAGTCTGTGCGGTTTTTGGA |
| Cbr4 reverse | ACATCACACCTAAATGCCAAGTG |
| Actb forward | GTGACGTTGACATCCGTAAAGA |
| Actb forward | GCCGGACTCATCGTACTCC |
| GAPDH forward | GGAGCGAGATCCCTCCAAAAT |
| GAPDH reverse | GGCTGTTGTCATACTTCTCATGG |

**SupplementaryTable2 Antibody information**

| **Antibody** | **Company** | **Catalogue** | **Dilution ratio** |
| --- | --- | --- | --- |
| GAPDH | Proteintech | 60004-1-Ig | 1：5000 |
| HIF1α | CST | 36169S | 1：1000 |
| Ki67 | Proteintech | 27309-1-AP | 1：2000 |
| CBR4 | Proteintech | 13725-1-AP | 1：1000 |
| β-Actin | Proteintech | 60008-1-Ig | 1：5000 |
| mTOR | CST | 2983S | 1：1000 |
| Ser2448 mTOR | CST | 5536S | 1：1000 |
| FASN | CST | 3180S | 1：1000 |
| HA | Proteintech | 51064-2-AP | 1：1000 |
| Goat Anti-Mouse IgG | CWBIO | CW0102S | 1：5000 |
| Goat Anti-Rabbit IgG | CWBIO | CW0103S | 1：2000 |

**Supplementary Table3 Short hairpin targets**

| **Gene names** | **Target sequence(5’-3’)** |
| --- | --- |
| CBR4 sh1 | GCTGGAGAAACATTTAGGTCG |
| CBR4 sh2 | GCTGCCATGAGGACTATGATT |
| Cbr4 sh1 | ATTCAGCAGGGAGGGTCTATT |
| Cbr4 sh2 | GGCATTTAGGTGTGATGTTGC |
